# Detection of Early Disease Risk Factors Associated with Metabolic Syndrome: A New Era with the NMR Metabolomics Assessment

**DOI:** 10.3390/nu12030806

**Published:** 2020-03-18

**Authors:** Julia Hernandez-Baixauli, Sergio Quesada-Vázquez, Roger Mariné-Casadó, Katherine Gil Cardoso, Antoni Caimari, Josep M Del Bas, Xavier Escoté, Laura Baselga-Escudero

**Affiliations:** 1 Eurecat, Centre Tecnològic de Catalunya, Unitat de Nutrició i Salut, 43204 Reus, Spain; julia.hernandez@eurecat.org (J.H.-B.); sergio.quesada@eurecat.org (S.Q.-V.); roger.marine@eurecat.org (R.M.-C.); katherine.gil@eurecat.org (K.G.C.); antoni.caimari@eurecat.org (A.C.); josep.delbas@eurecat.org (J.M.D.B.); 2 Universitat Rovira i Virgili; Department of Biochemistry and Biotechnology, Ctra. De Valls, s/n, 43007 Tarragona, Spain

**Keywords:** metabolic syndrome, metabolism deregulation, molecular biomarker, prevention, metabolomics, nutritional habits, carbohydrate dysfunction, dyslipidemia, oxidative stress, inflammation, gut microbiota

## Abstract

The metabolic syndrome is a multifactorial disease developed due to accumulation and chronification of several risk factors associated with disrupted metabolism. The early detection of the biomarkers by NMR spectroscopy could be helpful to prevent multifactorial diseases. The exposure of each risk factor can be detected by traditional molecular markers but the current biomarkers have not been enough precise to detect the primary stages of disease. Thus, there is a need to obtain novel molecular markers of pre-disease stages. A promising source of new molecular markers are metabolomics standing out the research of biomarkers in NMR approaches. An increasing number of nutritionists integrate metabolomics into their study design, making nutrimetabolomics one of the most promising avenues for improving personalized nutrition. This review highlight the major five risk factors associated with metabolic syndrome and related diseases including carbohydrate dysfunction, dyslipidemia, oxidative stress, inflammation, and gut microbiota dysbiosis. Together, it is proposed a profile of metabolites of each risk factor obtained from NMR approaches to target them using personalized nutrition, which will improve the quality of life for these patients.

## 1. Introduction

Metabolic syndrome (MetS) is considered a multifactorial disease, which means that a cluster of risk factors associated with disrupted metabolism may influence in their development [1]. Multifactorial diseases are caused by different single factors but also by a combination of altered metabolic situations (genetic, environmental, physiological, metabolic, cellular, and molecular elements) that working together and extended over time eventually lead to a pathologic state [2,3]. However, these processes are not fully understood yet and MetS has emerged as a worldwide health concern in the recent decades, which prevalence is growing in parallel with the incidence of obesity, type 2 diabetes (T2D) or insulin resistance (IR). Thus, MetS is mainly attributed to changes in lifestyle that may impact genetic and phenotypic susceptibility [4,5,6]. Subsequently, the opportunity to prevent this disease is presented as a medical challenge for the whole facultative and research community. 

Nowadays, there is a lack of efficient tools to prevent the development of MetS, obesity and their metabolic disarrangements, which essentially includes carbohydrate and lipid metabolism, inflammation, oxidative stress, and gut microbiota [7]. Nevertheless, there are several lifestyle aspects that can be modified to prevent the development of these risk factors associated to MetS such as diet, nutritional habits, and physical activity [8]. However, nutrition is probably the most important adaptable factor that regulates the expression of genes involved in several metabolic pathways [9]. Thus, driven changes in diet and nutritional habits, known as personalized nutrition, have been increasing as a promising tool and are taking more relevance in society to control and prevent metabolic diseases [10].

The classical concept of personalized nutrition is assisted by genetic assessment through an analysis of single nucleotide polymorphisms (SNPs), which may provide useful information about the genetically programmed response of a subject to a given food or nutrient (nutrigenomics) [11]. The phenotypic traits are dynamic markers and hence, more appropriate for defining the effects of lifestyle variables on the organism (diet, nutritional habits, physical activity, daylight rhythmicity, etc.). Advances in omics technologies have led to the possibility of characterizing the metabolism of every subject from a holistic point of view, thus opening a wide array of possibilities for phenotypic characterization and providing a more accurate health assessment contributing to improve quality of life [8]. 

Recently, the concept of nutrigenomics has evolved to incorporate many integrative methods concerning high-throughput omics technologies such as genomics, transcriptomics, proteomics, metabolomics, metagenomics, and epigenomics [12] because the personalized nutrition based in nutrigenomics is limited. Ideally, the optimal personalized nutrition should be based in this modern concept of nutrigenomics, but is reasonless in a practical way due to the methodology high cost and the technical difficulties [13]. 

An alternative of the classical and modern concept of nutrigenomics is the study of the metabolomic profile [14]. An increasing number of nutritionists integrate metabolomics into their study design, nutrimetabolomics, achieving to be one of the most promising avenues for improving personalized nutrition [15,16]. Personalized nutrition can target small deviations of the metabolism associated with the risk factors, before the onset of the disease. When the disease is finally developed, the problem escapes the field of personalized nutrition and medical drugs administration are required. Therefore, there is a real need for an early detection of the slight changes on different metabolic parameters that combined triggers the disease development. At present, the lack of robust health status biomarkers for the principal clusters of MetS and obesity is a bottleneck that slows down the personalized nutrition in metabolomics [17]. This fact has been taken up by the scientific community. For example, the BIOCLAIMS project (FP7-244995) which has established the principles to obtain robust biomarkers for health status, or the PREVENTOMICS project (DT-SFS-14-2018-818318) which aims to use health biomarkers in applications for consumers. 

In order to introduce the advantages of metabolomics in the research of biomarkers, the common techniques used in metabolomics should be known. The two most common techniques used are nuclear magnetic resonance (NMR) spectroscopy and mass spectrometry (MS) hyphened to chromatographic techniques such as gas chromatography (GC), capillary electrophoresis (CE), liquid chromatography (LC), and ultra-high performance liquid chromatography (UHPLC). Each analytical platform has its own advantages and disadvantages, thus the choice of the platform principally depends on the objective of the study, the accessibility and expertise of the platform [18]. The NMR platform is proposed as an emerging tool for large-scale metabolomics studies in the personalized nutrition field. The NMR characteristics which make it a unique platform include its high level of experimental reproducibility, its simplicity in sample pre-processing and preparation, its capacity to handle diverse biofluids, its quantitative capabilities (with a high coverage and low detection limits [19]), and its utility in identifying unknown metabolites along with its non-destructive nature [20]. The inherent limitation of NMR is the low sensitivity compared to MS but there are emerging new NMR technologies that suggest a huge improvement in the NMR spectroscopy [21]. In order to highlight one of the NMR-approaches, quantitative proton ^1^H-NMR is the most useful NMR-based platform for metabolomics and has been successfully applied to early diagnostic and prognostic purposes [22,23]. The most popular biological fluids used in metabolomics are plasma, serum, urine and feces, while other fluids and tissues are not yet well explored. Plasma and serum are the most common biofluids used in human metabolomic studies, because they are relatively easy to collect with minimal invasive procedures and their metabolome reflects individual changes in metabolism. On the other hand, the advantages of urine and feces samples are that they are biological samples easy-to-access, which can be obtained using non-invasive procedures [15]. Between urine and feces, urine is preferable as biofluid because the NMR techniques are optimized for early disease detection [24].

Taking into account these necessities, the present review addresses the demand to have a list of the potential molecular markers obtained by NMR metabolomics to be targeted in personalized nutrition in plasma/serum and urine (Table 1). We propose five clusters of molecular markers associated with five of the most relevant risk factors associated with MetS and related diseases. Then we will discuss the involvement of new biomarkers in the early stages of MetS distributed in the following list of molecular clusters: carbohydrate metabolism, dyslipidemia, inflammation, oxidative stress, and gut microbiota dysbiosis. 

## 2. Carbohydrate Dysfunction

Carbohydrate metabolism dysfunction is highly related with IR and T2D, which represents approximately 95% of diabetes cases worldwide [83]. The standard clinical determinations of carbohydrate dysfunction include glucose and insulin determinations; HOMA-IR (homeostasis model assessment of IR) calculated by fasting plasma glucose and insulin levels; glycated hemoglobin (HbA1c) determination; and adiponectin and leptin levels, and the ratio of both, as hormones produced predominantly by adipocytes involved in carbohydrate dysfunction [84].

Fasting plasma glucose levels upper 7 mmol/L and fasting plasma insulin below 110 pmol/L are related to the carbohydrate metabolism pre-disease [85,86]. HOMA-IR, which is a widely accepted method to calculate IR state, determines the IR using the fasting glucose and insulin levels as described in different clinical guidelines [87,88], following the formula (HOMA-IR = Insulin (µU/mL) × Glucose (mmol/L)/22.5) [89]. A higher value of HOMA-IR corresponds to a more severe IR [90]. Additionally, the HOMA-B index has been used as a robust measure of beta cell function (HOMA-B = Insulin (µU/mL)/(Glucose (mmol/L) −3.5)) [89], as well as the QUICKI index (QUICKI =1/(Log Insulin (µU/mL) + Log Glucose (mmol/L)), which is considered a measure of insulin sensitivity [91]. As a conclusion, nowadays HOMA-IR is the most frequently used index to determine IR using fasting blood levels of glucose and insulin [92]. 

Other typical determination in T2D diagnosis is HbA1c, which was initially identified as an “unusual” hemoglobin, and has been correlated with glucose in several studies, suggesting the idea that HbA1c could be used as an objective measure of glycemic control [93]. HbA1c values represent the average glycemic control over the past 2–3 months and account for both, pre-prandial and post-prandial blood glucose levels [94]. Moreover, regular HbA1c measurement is recommended by different international guidelines for all patients with diabetes for the assessment of glycemic control [95]. Although the HbA1c concentration is used for diagnosis, the biological variation and non-standardized procedure limits its application [96].

In addition, some hormones secreted by the adipose tissue, such as the adipokines leptin and adiponectin, interact in modulating T2D risk, being adiponectin more strongly associated with T2D risk [97]. Specifically, the circulating levels of adiponectin are inversely associated with pre-diabetes and other metabolic traits [98,99,100]. In the case of leptin, higher circulating levels are directly contributing to the development of IR. Moreover, the leptin/adiponectin (L/A) ratio is related with preventive measures in MetS [101] and highly associated with IR in non-diabetic patients [102]. In the ARIRANG study, low ratio of L/A is a predictor for the regression of MetS and L/A was proposed as a clinical biomarker to measure the risk to develop the syndrome [103]. Finally, L/A ratio and HOMA-IR index has been demonstrated that both can be used to identify obese patients with IR [104,105].

Regrettably, insulin, HbA1c, leptin and adiponectin levels detected by traditional methods are only useful when the disease is well-stablished and not in the preliminary states of the pathology. Thus, we propose different metabolites, that are detected by NMR approaches, as molecular markers of carbohydrate metabolism dysfunction that can be detected in the pre-disease state. Specifically, we propose glucose and lactate as principal bioenergetics molecules and, new emerging biomarkers, such as plasmatic levels of uric acid; branched chain amino acids (BCAA); aromatic amino acids (AAA); other amino acids as glutamate and glutamine; or propionylcarnitine.

### 2.1. Glucose

Glucose is a classic carbohydrate used as a biomarker for the diagnostic for carbohydrate dysfunction metabolism [106]. In the absence of more specific biological marker to define T2D, glucose has been used as a marker for diagnostic criteria for T2D and pre-diabetes according to the 2006/2013 World Health Organization (WHO) [84,107] and 2019 American Diabetes Association (ADA) recommendations [108]. Carbohydrate metabolism is important in the development of T2D, where insulin regulates the blood levels of glucose and its metabolism helping cells to take glucose or store it as glycogen, depending on the needed. To sum up, high blood levels of glucose finally results in alteration of pancreatic β-cell function carrying on with IR [109]. Moreover, glucose, which is the primary source of energy for living organisms, could be broken down via glycolysis, enter into the TCA cycle and oxidative phosphorylation to generate nucleotide adenosine triphosphate (ATP). Other important pathways in carbohydrate metabolism are glycogenesis, glycogenolysis (conversion of glycogen polymers into glucose, stimulated by glucagon) and gluconeogenesis (de novo glucose synthesis) [110]. 

There are several pre-clinical and clinical evidences about the potential of glucose as early biomarker of disease using NMR method. However, it is difficult to identify other metabolites in samples with an imbalance of glucose because the glucose signals (including other metabolites that overlap with the glucose region) suppress the other metabolite signals in the NMR spectrum [111]. In animal studies, high levels of glucose are shown in the metabolic profile. For example, Abu Bakar Sajak et al. [26] and Mulidiani et al. [25] detected and quantified glucose in urine in streptozotocin (STZ)-induced diabetic rats. In recent clinical studies, glucose is significantly increased in adults with risk to develop MetS or related diseases [95]. In forty-six young adults of normal weight and overweight, the serum metabolite profile was analyzed by NMR and high levels of glucose were detected in overweight adults compared to normal weight volunteers [27]. Moreover, the importance of using glucose in the profiling of a pre-disease state was stablished in another clinical trial, where healthy people and patients with different levels of T2D presented an increase on glucose concentration depending on the disease state (T2D and its complications) [28]. In addition, Zhang et al [19] aimed to identify the biomarker signature of pre-states in metabolic diseases by serum profiling with NMR. Principal components analysis and orthogonal partial least squares-discriminant analysis were used to distinguish between samples from patients and healthy controls. In this study, glucose was highly expressed and included in the suggested metabolic profile for the early prediction [19]. However, taking into account the wide and easy extended use of glucometers to measure glycaemia, measure glucose levels with NMR analysis will be not justified unless additional parameters would be obtained in the same NMR profile.

### 2.2. Lactate 

Focus on carbohydrate metabolism dysfunction, lactate has been considered a disease biomarker but also a marker for the pre-disease stage [112]. It plays a role in several biochemical processes and it is also an end-product of bacterial fermentation, produced by lactic acid bacteria of the genera *Lactobacillus* and *Bifidobacterium* [113] (discussed below). Lactate is formed in mammalian cells predominantly from glucose and alanine through their conversion into pyruvate, which is reduced to lactate by lactate dehydrogenase. Besides, the same enzyme removes lactate via its oxidation to pyruvate. Pyruvate could be oxidized to carbon dioxide producing energy or transformed glucose. Lactate metabolism is directly implicated in the gluconeogenesis, indirectly in the TCA cycle and in the respiratory chain, which are metabolic pathways implicated in carbohydrate metabolism [114]. Changes in plasma lactate during an oral glucose tolerance test (OGTT) are inversely correlated with fasting insulin, indicating that IR can be reflected through this metabolite response to a glucose challenge [115,116]. Lactate homeostasis is related to glucose metabolism and, therefore, diseases associated with glucose disruption, as MetS, obesity or diabetes, are associated with disturbed lactate metabolism [114,117]. Failures in the mitochondrial energy-generating system in the pancreatic β-cells may also lead to the abnormal accumulation of lactate in urine, blood, and cerebrospinal fluids [118].

The first metabolomic approach to quantify lactate urinary level, which determined lactate as a risk marker for T2D, was done by Chou and their colleagues [31]. In animal studies, high levels of lactate were shown in urine NMR metabolomic profile in rats feed with a high fat diet (HFD) and in obese rats [29,30]. In these studies, lactate was selected as key metabolite in the carbohydrate’s disruption. In overweight volunteers, lactate was increased compared with normal weight patients in the serum metabolite profile [27]. In a longitudinal clinical study, OGTT was assessed in two Finnish population-based studies consisting of 1873 individuals and re-examined after 6.5 years. Metabolites were quantified by NMR from fasting serum samples and the associations were studied by linear regression models adjusted for established risk factors. Lactate was determined as potential marker for long-term IR that could be related to glucose tolerance later in life [32]. Consequently, changes in lactate levels are a promising tool to monitor early disarrangements in the carbohydrate metabolism.

### 2.3. Uric Acid 

Uric acid, generated during ATP metabolism, is the end product of the exogenous pool of purines and endogenous purine metabolism [119]. In the purine metabolism, adenosine monophosphate (AMP) deaminase promotes fat storage and IR, whereas activation of AMP activated protein kinase stimulates fat degradation and decreases gluconeogenesis. Uric acid is a key factor that appears to promote the mechanism implicated in imbalanced carbohydrate metabolism [120,121]. Overproduction of uric acid has been implicated in chronic diseases states including MetS, pre-diabetes, hypertension and non-alcoholic fatty liver disease (NAFLD) [122,123,124,125]. In addition, uric acid has been described as an antioxidant molecule [126] which will be discussed in the oxidative stress section. Elevated uric acid may become one of the most important molecular markers for early-phase mechanisms in the development of MetS and other metabolic diseases [127,128].

In pre-clinical studies, elevated serum levels of uric acid, determined by NMR approach, were found in STZ rats [34] and in obese mice [35], compared to control animals, associating this metabolite with diabetes and obesity. In a clinical work focused on an NMR-based metabolomic investigation of the serum profiles of diabetic, higher concentration of uric acid was detected in T2M subjects [36]. All these evidences place uric acid as a promising new biomarker for the early detection of metabolic alterations.

### 2.4. Propionylcarnitine

Acylcarnitines play an essential role in the regulation of carbohydrate and lipid metabolism balance. They are esters of L-carnitine and fatty acids formed in the cytosol to transport fatty acids into the mitochondrial matrix for β-oxidation as a major source of energy for cell activities. The involvement of acylcarnitines in the intermediary metabolism is essential to mammalian bioenergetics process, and it is needed for the carnitine-dependent production of energy from different fatty acids and for the cell membrane structure maintenance [129]. Disruption in fatty acid oxidation results in elevated acylcarnitine concentrations, suggesting that more fatty acids are entering into the mitochondria [130]. It has been described that concentrations of some acylcarnitines are associated with MetS, obesity, and pre-diabetes [131,132,133,134]. The mechanisms by which acylcarnitines contribute to mitochondrial dysfunction have yet to be fully elucidated [135].

The acylcarnitines, which have been related to a pre-disease state, are not clear markers but, among the different types of acylcarnitines, the propionylcarnitine (C3) is the most promising short chain acylcarnitine to become a pre-disease biomarker. In general, the levels of blood acylcarnitines inadequately reflect tissue acylcarnitine metabolism [37], but C3 is one of them overcoming this impediment. In some studies of short-chain carnitine esters, C3 has been positively associated with T2D risk and IR [136]. On the other hand, the combination of C3 with other metabolites of interest, such as BCAAs, glutamate/glutamine, and methionine, was particularly most robust to differentiate metabolically lean from obese patients [38,39]. In other clinical study, twenty-four acylcarnitines were measured in more than a thousand subjects which were grouped by normal glucose tolerance, isolated impaired fasting glycaemia, impaired glucose tolerance or T2D [40]. Serum levels of C3 stood out significantly among the groups, proving its relevance as a robust biomarker of early stages of carbohydrate metabolism disorders [40]. Finally, the accuracy of MS in acylcarnitine profile determination is the main reason why most of the studies analyzing acylcarnitines are performed by using this approach. In the latest years the NMR techniques have been improving to screen the acylcarnitine profile [137], what will allow to obtain a more precise vision of the early involvement of C3 in the development of metabolic diseases, and at the same time, to detect the contribution of other acylcarnitines in these critical phases that have so far gone unnoticed.

### 2.5. BCAAs and AAAs 

BCAAs (isoleucine, leucine, and valine) and AAAs (including phenylalanine and tyrosine) are essential amino acids; this means that they cannot be synthesized de novo by human cells, forcing to be obtained from the diet. Once inside the body, the levels of are relatively stable in blood and tissues (Table 2 shows normal levels of these amino acids). BCAAs and AAAs are mainly regulated by their catabolic pathways (which are mainly localized in the mitochondria of all tissues), then higher plasma levels of these amino acids are well correlated with several pathologies. Consequently, BCAAs and AAAs are potential biomarkers which have been shown to be associated with a ~5-fold increased risk of developing T2D [138,139].

BCAAs cluster has been more exploited as a health marker than AAAs in the literature. An overwhelming number of publications and multiple studies support that concentrations of BCAAs in plasma and urine are associated with IR [143]. BCAAs play an important role in the regulation of energy homeostasis, nutrition metabolism, gut microbiome health, immunity, and disease in humans and animals [144]. As the most abundant of essential amino acids, BCAAs are not only the substrates for synthesis of nitrogenous compounds, they also serve as signaling molecules regulating glucose, lipid, and protein synthesis [144]. Metabolomic profile of obese *vs.* lean subjects reveals a BCAA-related metabolite signature that is suggestive of increased catabolism of BCAAs and it is positively correlated with IR. The increased BCAAs was reported to stimulate gluconeogenesis and glucose intolerance via glutamate transamination to alanine [140]. In addition, BCCAs detection and quantification are highly correlated using both NMR and MS methods, becoming BCAAs as a suitable new biomarkers for disease prevention [145].

AAAs cluster has been less exploited but there is a real evidence of two amino acids, phenylalanine and tyrosine, implicated in the pre-disease stages. Both amino acids are involved, as BCAAs, in protein synthesis. Tyrosine is considered a semi-essential amino acid because it can be synthesized from phenylalanine, and both are the initial precursors for the biosynthesis of fundamental neurotransmitters or hormones in animals and humans [146]. BCAAs and AAAs have been related to MetS, obesity, and T2D in animal models and in human studies, both longitudinal and cross-sectional studies, having in common the usage of NMR metabolic profiles. For example, elevated levels of BCAAs and AAAs have been reported between diabetes and control group in STZ rat model [25,26]. In a longitudinal human studies, BCAAs were associated with higher glycaemia and IR and post-challenge glucose levels using NMR approach [32]. A recent meta-analysis of four groups of patients with pre-diabetes and diabetes showed that BCAAs were elevated by approximately 40% in the setting of poor glycemic control [41]. Moreover, BCAAs and AAAs were significantly different between metabolically healthy overweight/obese and MetS women, independent of other risk factors [43]. In other study of women transitioning from gestational diabetes mellitus to T2D, the BCAAs-related metabolite cluster was tightly associated with the incidence of T2D in the different groups [42]. 

Altogether, the studies using NMR approaches have reported an increase level of circulating BCAAs and AAAs consequently of the dysfunction of carbohydrate metabolism. Some studies include BCAAs and AAAs together representing a profile biomarker, and others only use a specific BCAA or AAA. For example, isoleucine and tyrosine were different between women who develop gestational diabetes and those who remained normal glucose tolerant [44]. Tyrosine was suggested as a particularly strong predictor of metabolic and obesity traits in South Asian individuals determined in a unique healthy cohort with follow-up during nineteen years by NMR approach between nine amino acids [48]. In some studies, valine stands out with an increase predisposition to develop T2D in the future. This fact is showed in the study of the relation between circulating metabolites and abdominal obesity in twin’s brothers [45] or the study revealing the predisposition to develop T2D in Chinese population [46]. Moreover, in 263 healthy men with MetS and their control counterparts, Siomkajło et al. proposed a diagnostic model consisted of phenylalanine as a marker obtained from omics technologies and other classical determinations [47]. 

All in all, the selection of the best option would be the utilization of BCAAs and AAAs as two clusters because the choice of one specific amino acid is controversial. There is a need of more robust studies using NMR methods to elucidate the implication of each amino acid in health metabolism and to elucidate a common outline. 

### 2.6. Glutamate Family: Glutamine and Glutamate

Besides BCAAs and AAAs, other common amino acids are potential biomarkers, such as glutamine and glutamate, of the dysregulation of carbohydrates metabolism. In recent studies, the profile of amino acids, including BCAAs, AAAs and glutamine and glutamate, has been linked with risk factors related to T2D [28,147]. In this section, plasma glutamine, glutamate and their ratio will be discussed as potential biomarkers for T2D as it is showed in several studies [148]. Glutamine and glutamate are key amino acids in the mammal intermediary metabolism and, they are also associated with aerobic metabolism via the TCA cycle and with ammonia metabolism [149]. 

Glutamine plays a crucial role in various cellular processes, such as in energy balance, apoptosis, and cell proliferation and, its deprivation can activate the fatty acid β-oxidation pathway [150,151]. For instance, there has been a controversy linking glutamine with the prediction of the T2D. An inverse association of glutamine with the risk of T2D has been hugely observed in the literature but some studies reported a positive association. This inconsistency was solved by Guasch-Ferré et al. after a systematic review. They concluded that the strongest association of glutamine is the inverse with the risk to develop T2D [50]. In a recent animal study, changed levels of glutamine were shown in HFD-fed rats compared with control group in urine NMR metabolomic profile [30]. 

Glutamate is produced in the first step of BCAAs catabolism [152]. Different authors have proposed that glutamate likely stimulates glucagon release from pancreatic α cells and increases transamination of pyruvate to alanine, which strongly promotes gluconeogenesis in obesity [153]. Thus, circulating glutamate is positively related to visceral obesity and posterior development of MetS [154]. In a pre-clinical study, IR was correlated with glutamate in mice treated with monosodium glutamate to develop obesity [49]. Moreover, in obese morbid patients, those with pre-diabetes were found to have higher serum glutamate levels compared to non-diabetic controls. It was speculated that glutamate was elevated in morbidly obese patients due to an increased need for α-ketoglutarate in the TCA cycle to compensate the IR. This same study also found that morbidly obese non-pre-diabetic group had increased levels of glutamate compared to non-obese and non-pre-diabetic groups, suggesting that obesity plays a role in glutamate metabolism [51]. As other amino acids, the detection and quantification of glutamine and glutamate by NMR methodology is evident and accessible as it has been shown. Therefore, they are promising metabolites for the prevention of carbohydrate metabolism dysfunction. 

### 2.7. Citrate

Currently citrate has been studied as a metabolite that could be a good biomarker to detect carbohydrate dysfunction [29,52]. Citrate is an intermediary of the TCA cycle, being synthetized from fatty acids and glucose, and it is regulated by glucose levels and insulin [52]. It is mostly analyzed in urine as a key metabolite contributing to the detection of metabolic disruptions. In a preclinical study, rats were fed with HFD or control diet and their urine was analyzed by NMR. The results showed higher levels of citrate in the HFD group. The authors also found differences between high gainers and low gainers. Thus, citrate variation is associated to diet and physical constitution, being higher in these animals with obesity and a gainer constitution [29]. E-Y, Won et al. also showed an increase on citrate levels in the urine of obese mice in comparison with control group, analyzed by NMR [52]. Corroborating this study, an increased levels of citrate were observed in other study of HFD-induced obese animals due to hyperglycemia and IR [71]. These alterations in different studies of citrate levels suggest a closed relation with the disturbances in glucose and insulin in obesity [52]. Inversely, it was reported a depletion of the citrate levels in urine associated with a higher level of IR in humans [53]. Furthermore, in obese and IR animals a decrease on citrate urinary levels was also observed, and the opposite result was seen in obese animals without IR [155]. More clinical studies using NMR approach should be done to have more information about the possibility of citrate as a biomarker of MetS. It is hypothesized that the increase on citrate concentration might be originated through increased free fatty acid (FFA) oxidation due to higher levels of FFAs. This oxidation cause an elevation of acetyl-CoA:CoA and NADH: NAD^+^ ratios in the mitochondria, where pyruvate hydrogenase is inactivated, rising the levels of citrate, which inhibits phosphofructokinase activity, causing an accumulation of glucose-6-phosphate. The glucose-6-phosphate may inhibit hexokinase II, decreasing glucose uptake [156]. Thus, citrate became a key player in the carbohydrate and lipid metabolism as well a potential new biomarker for the metabolic syndrome.

## 3. Dyslipidemia 

One of the main consequences of the MetS is cardiovascular disease (CVD), which remains as the leading cause of morbidity and mortality in the western countries and whose incidence is increasing daily mainly due to diet and lifestyle [157]. Numerous risk scores have been developed to predict CVD risk (Atherogenic Index of Plasma (AIF); Framingham and Reynolds scores; etc.) [158,159]. These scores are based on clinical observations of individual traditional biomarkers of serum lipids, glucose, and hormone profile [159]. Indeed, dyslipidemia is an abnormal amount of lipids in the blood that it is generally characterized by an elevation of triglycerides (TG), non-high-density lipoprotein-cholesterol (non-HDL-C), and low-density lipoprotein-cholesterol (LDL-C), and in parallel, a reduction in the high-density lipoprotein-cholesterol (HDL-C) [160]. In addition, dyslipidemia is also promoted in obesity, T2D and IR by a prolonged elevation of insulin levels. The association between obesity and CVD risk factors may be mediated by the ability of adipose tissue to synthesize and secrete several hormones with a systemic influence, including leptin and adiponectin. Leptin plays an important role in the regulation of feeding behavior and their levels reflect the amount of energy reserves stored in adipose tissue [158]. On the other hand, adiponectin levels are inversely associated with body fat mass, inflammation, dyslipidemia, T2D, and MetS; and their levels may be increased by healthy dietary patterns [161]. However, conventional algorithms to detect CVD risk factors are stablished in diseased population [159] and not in the preliminary stages of disease. 

Unfortunately, these traditional biomarkers are not enough to evaluate the disease progression and status of emerging risks in apparently healthy patients. Hence, other biomarkers, alone or in combination, should be incorporated into risk prediction models to determine whether their addition increases the model’s predictive accuracy and reliable estimation of CVD risk related to dyslipidemia. Thus, an early identification and treatment of risk factors are much needed to accelerate disease prevention and morbidity improvement. Consequently, in the absence of disease and, therefore, without pharmaceutical treatment, the robustness of this prediction model will allow to reduce the potential cardiovascular risk by acting on specific dyslipidemia cluster using precise nutritional recommendations.

### 3.1. Fatty Acids: Saturated, Monounsaturated, and Polyunsaturated 

Lipid and carbohydrates metabolism are closely interconnected. In fact, altered fatty acid profile affects IR and T2D; and vice versa [162]. Structurally, fatty acids can be split by the presence of double bounds in their backbone as saturated (SFA; absence of double bound) and unsaturated fatty acids [162]. Unsaturated fatty acids can be further divided by the number of double bounds as mono- (MUFAs; a single double bound) and poly-unsaturated fatty acids (PUFAs; more than one double bound) [163]. SFAs, MUFAs and PUFAs present different biological properties. The types of fatty acids present in various food groups are thought to play a pivotal role in whether or not such food is considered beneficial, neutral, or detrimental with respect to developing MetS and related diseases. It is well established an implication of dietary fats as risk factors for T2D and MetS, especially for long chain SFA (C14:0, C16:0 and C18:0) which could induce IR, whereas increased circulating levels of very long-chain SFA (C20:0, C22:0 and C24:0) are associated with reduced T2D risk [164,165]. In the case of palmitate, its presence activates receptor FFA from beta-cells initiating a cascade with cell stress responses as ceramide formation, lipid droplets formation, endoplasmic reticulum stress, mitochondrial dysfunction and autophagy triggering an impairment of insulin secretion and damage in beta-cells [166]. PUFAs include some subgroups identified by the position of the last double bond in their molecular structure [164]. PUFA *n*-3 include mainly alpha linoleic acid (ALA), eicosapentaenoic acid (EPA) and docosahexaenoic acid (DHA), while PUFA n-6 include linoleic acid (LA), and arachidonic acid (AA) [162]. Thus, both MUFA and PUFA have been related to an improvement of insulin sensitivity. 

Numerous beneficial healthy effects have been attributed to unsaturated fatty acids, including protection from obesity, diabetes, cancer, and atherosclerosis [162,164]. The most abundant MUFA in typical diets is oleic acid (C18:1*n*-9) which is effective in lowering the inflammatory response and LDL levels; that together contribute in the reduction of CVD risk [162,163]. High levels of MUFAs were described in the prevention of abdominal fat accumulation. Moreover, the substitution of carbohydrates with MUFA cause a decrease on total blood cholesterol an TGs, reducing the levels of HDL-C [162,163]. The mechanism involved in the anti-inflammatory effect of MUFAs is the inhibition of NF-kB activity [167]. In an animal study, Guo et al. demonstrated with NMR that high fat fed animals presented a significant increase on TG, LDL/VDL and SFAs levels and a decrease in the PUFA/MUFA ratio [168]. However, in a NMR study of hundred three obese women divided by the absence or presence of MetS, several species of PUFAs were associated with MetS [70]. In addition, different studies have been done to associate PUFAs with inflammatory parameters. EPA and DHA have been seen to exhibit anti-inflammatory properties and are also important to produce eicosanoids from the *n*-6 fatty acid like arachidonic acid [43]. Thus, even the generally beneficial effects attributed to PUFAs, deeper research is necessary to identify the relevance of every fatty acid species levels in the context of dyslipidemia as in the development of metabolic and CVD.

### 3.2. 3-Hydroxybutyrate

Acetoacetate, 3-hydroxybutyrate (3-OHB) and acetone are ketone bodies, emerging as crucial regulators of metabolic health and produced in the liver from fatty acids that serve as a circulating energy in situations of glucose deprivation (i.e. fasting, carbohydrate restrictive diets, prolonged intense exercise, and ketogenic diets) [169]. Ketone bodies have a characteristic smell, which can easily be detected in the breath of persons in ketosis and ketoacidosis [170]. 3-hydroxybutyrate (or β-hydroxybutyrate) serum levels can increase thousands of times in their concentrations after a prolonged fasting and present a broad range of signaling and regulatory effects including inhibition of many deacetylases [170]. Moreover, 3-hydroxybutyrate is described to induce resistance to oxidative stress via deacetylases inhibition that may explain, at least partially, the therapeutic value of low-carbohydrate and ketogenic diets [170]. Mitochondrial β-oxidation of FFAs results in the production of Acetyl-CoA, which might go into the TCA cycle for further oxidation. Acetyl-coA is condensed to ketone bodies in the liver by ketogenic enzymes, for example 3-OHB [171]. Bugianesi et al. [172] found in NAFLD patients increased 3-OHB circulatory levels associated with hyperinsulinemia. Taken together, application of new diagnostic tools based in NMR will contribute to understand the uses of 3-hidroxybutirate as biomarker for MetS. Thus, hamsters fed with high-fat high-cholesterol diet showed an increase in the urine levels of 3-hydroxybutirate [173]. These results were corroborated at serum level, observing that high fat–fed mice showed an increase in 3-hydroxybutirate concentration [174]. Similar results were observed at plasma, where T2D patients presented increased 3-hydroxybutirate levels [71]. Therefore, these evidences point 3-hydroxybutirate as an important biomarker to taken in consideration for an early metabolic disarrangements’ detection.

### 3.3. Choline

Choline is an essential nutrient for maintaining human health which is involved in the mobilization of fat from liver [72]. In animals, the 95% of the total choline in tissues is used for the formation of phosphatidylcholine (PC) via the Kennedy pathway. It was described circulatory PC levels were increased in high-fat diet fed animals. PC is essential for the packaging, exporting and secreting of TG in VLDL, and acts as an intermediary to maintain a balance between fat in plasma and in the liver [175]. Choline deficiency results in various disorders, as fatty liver and liver dysfunction, which leads to elevations in serum concentrations of the liver aminotransferases [27]. Moreover, choline is a precursor of the neurotransmitter acetylcholine and it is essential in the membrane phospholipids and lipoproteins structure [175]. Consequently, it performs important functions in signal transduction, neurotransmitter synthesis or lipid transport. Moreover, plasma choline levels exhibited a positive correlation with serum TG and glucose levels, showing its involvement in the pathogenesis of several diseases, including MetS, fatty liver, obesity, or cardiovascular disease [175]. Indeed, monkeys fed with high fat and high cholesterol diet showed lower serum level of choline and an inverse correlation with TG levels, explaining the relation between the lack of choline and the accumulation of TG in the liver [74]. A clinical study based in the differences between overweight patients and control subjects about metabolites levels. In the case of choline, was decreased in overweight patients in comparison with healthy subjects, showing a relationship between choline and a disruption in the lipid metabolism [73]. Although these evidences, more studies with NMR are necessary to decipher the specific contribution of choline as a new biomarker for the early MetS detection.

## 4. Inflammation 

Obesity and MetS are described as risk factors for T2D and CVD, which are viewed as inflammatory diseases. One of the main causes of chronic inflammation is the constant overload of glucose and FFAs, that promote the production of pro-inflammatory signals or elevates reactive oxygen species (ROS) levels. Chronic inflammation and immune cells are related to the pathogenesis of IR in obesity [27]. The best described markers of inflammation are cytokines released by immune cells, C-reactive protein (CRP) and monocyte chemoattractant protein 1 (MCP-1), interleukin (IL)-6, IL-8, or tumor necrosis factor α (TNFα) [176]. As it happens with other common biomarkers, these cytokines are analyzed by ELISA methods, which are time-consuming, labored and less reproducible in comparison with NMR analysis. Therefore, to extract levels of other reliably biomarkers of inflammation from the NMR profiles it would be beneficial for the early detection of metabolic alterations. In this section we will focus on the role of n-acetylglycoproteins and lysophospholipids in the inflammation cluster, but other metabolites such as PUFAs (including EPA; DHA; or ARA) also develop important inflammation roles as pointed in previous sections. 

### 4.1. N-Acetylglycoproteins 

Glycosylation is one of the most common post-translational modification of secreted proteins and their misregulation is related with inflammation and multiple diseases (CVD, T2D, cancer, etc.) [177,178]. Therefore, human glycome is a novel tool to identify biomarkers and potential mechanistic mediators of pathogenesis. Indeed, increased serum glycoproteins levels are positively correlated with CRP levels [179]. The interest to study glycans, as an early biomarker of disease, is due to an altered glycosylation pattern might reflect the development of diseases [66]. Lawler et al. identified a glycoprotein-N-acetyl methyl group signature measured by NMR (GlycA) associated with CVD and T2D [66]. The two major contributors of the GlycA signal are α1-acid glycoprotein and haptoglobin, synthesized and secreted by neutrophils granules, as well by the liver [66]. The potential risk associated with elevated GlycA would relate to activation of systemic inflammatory pathways, because GlycA identifies aggregates of glycan moieties on circulating glycoproteins, which the majority of them are acute phase reactants and immunologic proteins [62,180]. In a large study with apparently healthy individuals, CVD mortality was significantly associated with elevated levels of GlycA [66]. The development of IR and β-cell dysfunction is triggered by low grade chronic systemic inflammation. Increased circulating levels of acute phase reactants are related to clinical expression of T2D, but is still unknown whether GlycA will be a proper marker for early detection of disease development [66]. The first evidence for the potential role of GlycA as a biomarker predictor in development of T2D [181] was described by 26,508 apparently healthy women, providing evidence of the potential role of glycans in the development of the disease. Moreover, it was suggested that elevated high GlycA might be correlated with a chronic inflammatory state [65]. Bervoets et al. [67] studied the plasma metabolic profile of obese children with NMR, and found N-acetyl glycoprotein increased in obese children in comparison with healthy children, and it could be traduced in an activation of the hexosamine pathway related to lower levels of glutamine and glucose. These proofs pointed GlycA as a better biomarker option for a systemic inflammatory response compared to traditional inflammatory cytokines, which often exhibit high intra-individual variability. Therefore, GlycA integrates the protein levels and glycosylation states of the most abundant acute phase proteins in serum [182], allowing a more stable measure of inflammation with lower variability. 

### 4.2. Lysophospholipids

Lysophospholipids are molecules derived from the hydrolysis of phospholipids, which transport fatty acids, phosphatidylglycerol, and choline between different tissues [183]. They are signaling molecules which modulate processes such as insulin production, insulin sensitivity and inflammation through interactions with G protein-coupled receptors [184], and are related to fatty liver, steatohepatitis, diabetes and obesity [184]. Different lysophospholipids species, mainly lysophosphatidilcholines (LPCs), have been identified as being altered in the plasma of obese individuals [185]. Significant amounts of circulatory levels of LPCs are synthetized by a specific enzyme activity lecithin, and lipoprotein-associated phospholipase A2 (Lp-PLA2), an inflammatory marker which has pro-inflammatory properties hydrolyzing oxidized phospholipids generating LPC under inflammatory conditions [186]. LPCs activate signaling pathways promoting the release of second messengers, related to G protein-coupled receptors [186]. In obesity, significantly lower concentrations of most of the LPCs are detected [68], whereas LPCs concentrations were inversely correlated with the increased CRP levels [184]. Therefore, LPC could be useful early biomarkers to detect inflammatory states associated with MetS and related disorders. 

## 5. Oxidative Stress

Oxidative stress appears as a risk factor when an imbalance of homeostasis happens between oxidant and antioxidant agents. The oxidant agents, mainly ROS and reactive nitrogen species (RNS), are constantly produced in the aerobic organism by normal metabolic processes (cellular respiration, antibacterial defense, etc.) and external exposures (smoking, toxins, ionizing radiation, etc.). In order to regulate the reactive species, organism has endogenous antioxidant systems, or it obtains exogenous antioxidants from diet, that neutralizes these species and keeps the homeostasis of the body [68]. Production of free radicals and the resulting oxidative stress are part of the energy metabolism, emphasizing mitochondrial dysfunction in the development of disease. Finally, the oxidative stress accumulation leads to the development of pathological condition as MetS, obesity and diabetes [187]. The inference of oxidative stress in T2D is done by the alteration in enzymatic systems, lipid peroxidation, dysfunction in glutathione metabolism and decreased vitamin C level [188]. The recommended biomarkers for monitoring oxidative status over time are 8-hydroxy-2′-deoxyguanosine (8-OHdG), F2-isoprostane 8-iso-prostaglandin F2α (8-iso-PGF2α), 3-nitrotyrosine, malondialdehyde (MDA), and oxidized low-density lipoprotein (oxLDL) [189]. These determinations are performed by ELISA kits. One of the most used is 8-iso-PGF2α, which are products of free radical-mediated oxidation of arachidonic acid. It has been detected to be altered in T2D, hypercholesterolemia, hypertension and MetS. The main biofluid in where it is determined is urine [190]. Other widely used is 8-OHdG, which represents the oxidative DNA damages [191]. However, the most popular determinations in plasma are 3-nitrotyrosine and MDA. 3-nitrotyrosine, the main product of tyrosine oxidation, has been described as a stable marker of ROS/RNS stress in inflammatory related diseases [192]. MDA, a small reactive aldehyde end product of the lipid peroxidation pathway, is a frequently used biomarker that can also be determined in urine, or tissue as thiobarbituric acid-reactive (TBAR) material, but the method is unstable and non-specific [193]. The last determination considered as classical is the oxLDL, which is the quantification of the oxidized LDL-C, but it is not stable in samples stored longer than a month [194]. All these determinations until the date are performed with expensive ELISA kits and sometimes the fine-tune determination depends on the storage time. Thus, it is essential to find different metabolites determined by NMR methods as new potential biomarkers in the risk factor of oxidative stress as allantoin, pseudouridine, and finally GSH (reduced glutathione)/GSSG (oxidized glutathione) ratio, glycine and serine as metabolites of the one-carbon metabolism. 

### 5.1. Uric Acid and Allantoin

Uric acid is accepted as the major antioxidant in plasma that protects cardiac, vascular, and neural cells from oxidative injury [195]. Uric acid, despite being a major antioxidant in the human plasma, it correlates and predicts positively and negatively the development of obesity and related diseases, conditions associated with oxidative stress and carbohydrate metabolism disruption as it is described in its section. Sautin and Johnson [196] tried to explain the paradox proposing that uric acid may function either as an antioxidant (primarily in plasma) or pro-oxidant (primarily within the cell). Therefore, considering the duality of the uric acid as a biomarker, we propose the end product of the uric acid oxidation from purine metabolism which is the allantoin as an alternative biomarker to uric acid [126]. Allantoin has been considered an oxidative stress biomarker as it also can be produced through non-enzymatic processes, especially when the levels of ROS are elevated [197]. While uric acid is considered antioxidant, allantoin is considered a pro-oxidant agent [198]. Urinary allantoin has been validated in a clinical model of oxidative stress, standing out its stability over different storage conditions as an oxidant biomarker [199].

There are several animal studies that determined allantoin as a biomarker in pre-disease using NMR metabolomic approach. In STD-rats, allantoin levels in urine stand out, among other metabolites, in T2D and obesity risk factor [196]. In a project characterizing biomarkers associated with T2D in eighteen biological matrices in *db*/*db* mouse model, allantoin was elevated in urine and plasma [26]. In other study characterizing the urine metabolome between lean and overweight dogs during a feed-challenge, overweight dogs had higher postprandial allantoin concentrations compared with lean dogs [76]. However, there is a need for more studies in humans and NMR approaches, because the evidence of the association in several animal studies should be confirmed with clinical studies. To date, only one study determined allantoin as a biomarker in humans, which aimed to predict gestational diabetes development using MS approach. This study showed higher levels of allantoin in the group of women with higher risk to develop diabetes [77].

### 5.2. Pseudouridine

Urinary excreted nucleic acids catabolites are used as non-invasive markers for oxidative processes related to resting metabolic rate and energy intake: 8-OHdG represents oxidative stress to DNA (considered a classical biomarker) and pseudouridine, the metabolite considered as a potential metabolic biomarker, determines oxidative stress to RNA [200]. Pseudouridine is an isomer of the nucleoside uridine in which the uracil is attached via a carbon-carbon instead of a nitrogen–carbon glycosidic bond. It is the most prevalent of the over one hundred different modified nucleosides found in RNA, being a marker of RNA degradation and damage in oxidative stress [201].

The trace of pseudouridine in NMR metabolic approaches had not been precise enough but nowadays there are promising studies in pseudouridine. For example, in a NMR metabolomics study trying to optimize quantitative urine metabolomics, urine and plasma samples from 1004 individuals correlated high levels of glucose and circulating amino acids with pseudouridine [202]. In a randomized controlled trial of VSL-based intervention (unknown product due to industrial interest) *vs.* control in children obesity complication leading to NAFLD, the pseudouridine was identified as a potential non-invasive metabolic biomarker by a urinary NMR metabolic profiling. Pseudouridine decreased in the VSL *vs*. the placebo group, concluding that pseudouridine may be increased in metabolic diseases as an oxidative risk factor [78].

### 5.3. One-Carbon Metabolism Intermediates: GSH/GSSG Ratio, Glycine, and Serine 

One-carbon (1C) metabolism is associated with metabolic disease, overweight, and obesity; higher levels of metabolites implicated in 1C metabolism are shown in healthy individuals [80]. The 1C metabolism consists on the transfer of one-carbon group and also, it is implicated in redox defense. The 1C metabolism is a reliable source of potential biomarkers as the selected, which are GSH/GSSG ratio, glycine and serine; thus, there are other with high probability to consider as betaine, dimethylglycine, methionine or cysteine [203]. One handicap to detect biomarkers of oxidative stress is the perception of oxidized metabolites, because the redox reactions could change the state of the metabolite (oxidized/reduced) during the manipulation of the sample and the redox ratio is difficult to determine. The GSH/GSSG ratio, which is an example in the 1C metabolism as an indicator of cellular health, is composed principally of reduced GSH constituting up to 98% of cellular GSH under normal conditions. The total quantification could be performed but the redox ratio calculation leads to more technical complications [204]. 

In order to avoid the problems in the determinations of redox ratio, an alternative to GSH/GSSG ratio is the selection of other metabolites of 1C metabolism. Glycine and serine, which are key amino acids in 1C metabolism, are proposed as a potential alternative to classical biomarkers [205]. For one hand, chronic glycine deficiency may impact health status, because glycine was found to have a strong negative association with IR when measured as HOMA-IR score [204], or by other methods (hyperinsulinemic/euglycemic clamp) [140]. This amino acid of lowest molecular weight, incorporates a hydrogen atom as a side-chain [206]. Glycine is a precursor for many pathways as glutathione synthesis, which has been related with oxidative stress as the master antioxidant, but it participates in other metabolic processes being an unstable measure to detect the risk factor of interest [204]. Some glycine derivatives have also been found to be associated with IR and the risk of T2D, one of them with the strongest relation is serine. Serine and glycine are very related. Loss of the mitochondrial pathway, renders cells dependent on extracellular serine to make 1C units and on extracellular glycine to make GSH [207,208].

There are some studies standing out some metabolites implicated in the 1C metabolism related to oxidative stress and metabolism disorder by NMR approaches. Specifically, serum glycine and serine were found in lower concentrations in participants with more MetS risk factors and greater adiposity, using modifiable lifestyle factors to attenuate health effects of obesity [209]. Further, plasma glycine and serine level were lower in obese diabetic African-American women compared to obese non-diabetic African-American women [81].

## 6. Gut Microbiota Dysbiosis 

100 trillion microbes exist in a symbiotic relationship with human cells, and the metabolic state of the human is related, in many cases, with the composition of the gut microbiota [82]. Numerous studies have shown that the gut microbiota composition may differ between lean and obese individuals or between pre-diabetic, T2D and normoglycemic individuals [210]. Dysbiosis of the gut microbiota, which is an alteration of the bacterial intestinal composition, reflexed a decreasing number of species related to an increased intestinal barrier permeability, thus allowing the bacterial translocation and causing endotoxemia [211], which is an important risk factor for obesity development and related metabolic diseases, as it is confirmed in different studies [212]. The constant flow in the composition of the gut microbiota is due to changes in diet, environmental factors and lifestyle [213]. As an intrinsic factor, the immune system health may cause changes in gut microbiota composition that may promote the proliferation of specific bacterial species which could be harmful due to the immune deficiency or hyperimmunity [214]. Genetics, age, or gender are factors that also affects in the human homeostasis [214]. The decrease of microbial diversity is triggered by different factors, such as the psychological stress, the type of diet or the higher sedentary lifestyle, causing dysbiosis [203]. Thus, this altered gut microbiota metabolizes different molecules, spreading metabolites in the blood, urine or feces which would be detected and used as biomarkers [215].

### 6.1. Lactate

Lactate, as it has been mentioned before, independent of participating in several biochemical processes is also an end-product of bacterial fermentation [216], produced by lactic acid bacteria of the genera *Lactobacillus* and *Bifidobacterium* [113]. Lactate is an intermediate metabolite, such as succinate, from the carbohydrate fermentation of some bacterial species. Moreover, it contributes to the maintenance of diversity within the colonic microbiota and the synthesis of the principal short chain fatty acids (SCFAs) [113]. Lactate is not accumulated in colon of healthy subjects, although a big proportion of intestinal bacteria can synthetize this metabolite, which is metabolized in butyrate or propionate [217]. In NAFLD patients was studied the composition of gut microbiota and selected bacterial products related with the fermentation of SCFAs in serum and feces by NMR analysis. The results showed higher levels of lactate in NAFLD patients, compared to control individuals, which was associated with reduced abundance of several bacterial species (*Ruminococcus, Coprococcus,* and *F. prausnitzii*) [217]. The amount and type of products can vary depending on species [33]. If the number of bacteria which metabolize lactate is decreased, excessive lactate production could end in its accumulation in the colon, where the absorption of lactate is low, lowering colonic pH and inhibiting the activity of microorganisms that metabolize lactate, for example propionate-producing bacteria or butyrate-producing. Butyrate is an inhibitor of acetate synthesis and the main energy source for colonocytes, could prevent the accumulation of lactate, which could be a potential toxic metabolite [33].

### 6.2. Acetate

Acetate, together with butyrate and propionate, is one of the three most common short chain fatty acid (SCFA) [218]. It is derived from intestinal microbial fermentation of dietary fibers in the colon [219] and acts as signaling ligand between host metabolism and the gut microbiome at different levels [220]. Acetate contribution leads to energy harvest participating in the human energy balance, with an important role in lipogenesis, cholesterol synthesis and accumulation in adipocytes [221]. Acetate affects substrate metabolism and host energy via an increase in energy expenditure and fat oxidation [222]. Via cross-feeding mechanisms branched-chain and aromatic amino acids might be produced and further metabolized, altering gut integrity and impairing insulin sensitivity. That is to say gut-derived acetate production is determined by the balance in gut between saccharolytic and proteolytic fermentation which is determined by the presence of acetogenic fibers [223]. *Firmicutes* are positive related to acetate, thus when dysbiosis cause an increase of *Firmicutes* in obese rats, plasma acetate levels increase, and it is linked to insulin action in morbidly obese individuals through circulating acetate. Fat cells release leptin in higher concentration by the presence of acetate [223]. In a human study with thirty-four morbidly obese women and men through NMR analysis, increased plasma levels of acetate were found, with a positive correlation with gut *Firmicutes*, and negatively correlated with HOMA-IR and fasting TG [223,224]. In a study with NAFLD patients, acetate was found increased in circulatory level and fecal level, analyzed by NMR. This increase was correlated with the reduction of the abundance of several bacterial species as *Ruminococcus, Coprococcus,* and *F. prausnitzii* [58]. HFD-induced obesity and IR in rats is associated with increased plasma concentration of acetate metabolized by the gut microbiota measured with GS-MS [33]. Less than 0.005% of the SCFAs were excreted into urine because they are excreted via the lungs after oxidation, that is why acetate is mostly identified in blood and feces [225]. Zang et al. studied female rats with diabetes by NMR urine analysis. It described acetate increased in diabetes group against control groups. The increase in the levels of acetate was correlated with higher levels of ethanol, and that suggested that the origin of this metabolites could be from microbial production, as in the case of *K. pneumoniae* [226].

### 6.3. Succinate

Succinate, a metabolite produced in the human body but also by the gut microbiota, is described as the major intermediary in the citric acid cycle, where it stands between succinyl-CoA and fumarate in the carbohydrate metabolism but the gut-microbiota produced succinate is classically described as an intermediate of the propionate synthesis [57]. Succinate has been increased in hypertension, ischemic heart disease, and T2D, but also in obesity, which is associated with elevated plasma levels of succinate concomitant with impaired glucose metabolism [227] Alterations in circulating succinate levels were associated with specific metagenomics signatures linked to energy production and carbohydrate metabolism [64]. It has been related with an antilipolytic action in adipose tissue through the succinate receptor 1 (SUCNR1), inhibiting the release of fatty acid from adipocytes. Thus, succinate has been related to cardiovascular diseases and obesity. In humans is found a strong association between microbial community, gene composition, and metabolism and plasma levels of succinate. In a study of a cohort of ninety-one patients stratified according to obesity and T2D, plasma succinate levels, analyzed by NMR and LC-MS, were significantly higher in obese than in lean individuals. A positive association was found between plasma levels of succinate and BMI, but also glucose, insulin, TG and HOMA-IR [64]. This increase in circulating succinate levels was associated with specific changes in gut microbiota related to succinate metabolism. *Prevotellaceae* and *Veillonellaceae*, succinate-producing bacteria, increased their relative abundance level in obese individuals. On the other hand, *Odoribacteraceae* and *Clostridaceae*, succinate-consuming bacteria, decrease their relative abundance level in obese individual. A significant increase of glycaemia was presented in these patients who present high circulatory levels of succinate, related to changes in gut microbiota associated to higher barrier permeability. Therefore, it explains the association of succinate as a microbiota-derived metabolite with an important role in obesity and metabolic-associated cardiovascular disorders [64]. It is also described a study with diabetic mice analyzed by NMR and the result was an increase of the succinate levels in urine [64]. Succinate has seen increased in fecal NMR analysis in NAFLD patients correlated to decreasing abundance of *Ruminococcus, Coprococcus* and *F. prausnitzii* bacteria in comparison with healthy individuals [63].

### 6.4. TMAO, TMA, and DMA

Trimethylamine (TMA) and trimethylamine N-oxide (TMAO) are metabolites which come from the choline metabolic pathway and L-carnitine [33]. Choline deficiency, which might cause microbial dysbiosis, is modulated by the conversion of dietary choline in TMA by gut bacteria, reducing the bioavailability of choline to synthesize phosphatidylcholine [228]. This TMA is released in the liver and is transformed in TMAO by the enzyme flavin-containing monooxygenase 3 (FMO3) [62]. These metabolites are seen to be related to the development of metabolic diseases, modulating the glucose metabolism in the liver and causing obesity [229], triggering inflammation in the adipose tissue and influencing lipid absorption and cholesterol homeostasis [230]. The fundamental role of the microbiota is evidenced in TMA production is derived from germ-free mice, which do not excrete TMA [231]. Using the urine of obese mice analyzed by NMR, TMA reflects metabolic changes related to HFD that follow body fat deposit [232]. An et al. studied the metabolic changes in HFD rats by NMR fecal analysis. HFD rats showed a level reduction of fecal TMA, which its origin is mostly from gut microbiota, probably resulted from its transportation to the liver, where is transformed in TMAO [59]. TMA showed positive correlation with gut microbiota from the genera *Allobaculum* and *Clostridium* [60,61].

TMAO present in urine and plasma is considered a biomarker for NAFLD, IR, and CVD [61]. Large perturbations in TMAO levels may result from dietary differences, and intestinal microbiota are suggested as playing a prominent role in the variation of TMAO levels. Some studies reinforced the importance of diet and microbiota in cardio-metabolic health, with the TMAO level emerging as a possible target for therapeutic interventions. Given that CVD risk in humans is linked to circulating levels of TMAO [233], and dietary supplementation with TMAO promotes atherosclerotic CVD in mice [234], a key opportunity for therapeutic research leads to blocking the ability of plasma TMAO to obtain a biological response. More than five hundred Finnish men with MetS was studied, the serum obtained was analyzed by NMR, and the results described a positive correlation between plasma TMAO concentrations and gut microbiota *Prevotella* and *Peptococcaceae*, however a negative correlation with *Faecalibacterium prausnitzii* was detected. These correlations are linked to dysbiosis in human disorders, such as obesity and diabetes [54]. A study analyzed by HPLC in diabetic patients, high levels of TMAO were found as a strong marker of all cardiovascular events, like in diabetic patients who tend to have elevated TMAO plasma levels. Thus, diabetes disease accentuates the relationship of elevated levels of TMAO and increased cardiovascular risk [56].

Dimethylamine (DMA) is also a metabolite generated from the TMA absorbed in the liver. High plasma and/or urine levels of DMA was described to be related to HFD induced IR, fatty liver, and T2D in mice [235]. In a mice study compared urinary metabolites of gut microbiota between HFD mice and control mice, this product of dietary choline processing by gut microbiota had a statistically significant result by NMR, showing a significant reverse correlation with total body fat. Thus, DMA could be considered a possible prospective biomarkers indicative of accumulation of body fat in obesity, being converted by the host liver to TMAO [62]. 

## 7. Relation between the Proposed Metabolites and Related Metabolic Pathways

To further characterize the metabolic pathways affected by the proposed metabolites, the metabolites were first annotated with Kyoto Encyclopedia of Genes and Genomes (KEGG) [59]. Then, the MetaboAnalyst (v4.0) software was used for metabolic pathway analysis and interpretation [236]. Eight pathways were statistically affected (FDR < 0.05) by the proposed profile of metabolites (see Table 3), thus five pathways stand out with high impact: the aminoacyl-tRNA biosynthesis; the glyoxylate and dicarboxylate metabolism; the alanine, aspartate and glutamate metabolism; the phenylalanine, tyrosine, and tryptophan biosynthesis; and the D-Glutamine and D-glutamate metabolism. These five pathways with high impact mainly affect amino acid biosynthesis and metabolism, except the glyoxylate/dicarboxylate metabolism and the aminoacyl-tRNA biosynthesis. Besides being involved in the different discussed clusters in the review, the metabolites proposed as early biomarkers for MetS are closely related to amino acid pathways and protein synthesis, suggesting that amino acid metabolism and associated pathways may be fundamental to the biologic processes that may underline prevention of MetS and associated diseases [237]. 

## 8. Future Perspectives

In this review, the detection of early molecular biomarkers has been highlighted as a promising strategy to prevent the development of MetS. Indeed, the finding of alterations in these metabolic parameters, which are closely related with robust clinical biomarkers such as glucose, triglycerides and cholesterol through several signaling pathways, could avoid the deregulation of metabolic pathways directly related with the development of MetS. However, the analysis of the described biomarkers would be relevant not only for the prevention of this multifactorial disease, but also for a large number of diseases, as there is a complex crosstalk between most of the metabolic parameters described in this review and several diseases, such as cancer, diabetes and neuro-related diseases. 

As an example, the gut microbiome product TMAO has been considered a shared risk factor between numerous diseases, such as IR, cancer, Alzheimer’s disease (AD) and schizophrenia, among others [238,239]. Clinical studies have described that higher circulating levels of TMAO are correlated with a higher inflammatory response (↑ C-reactive protein, ↑ TNF-α, ↑ IL-6) [240]. Moreover, it is related to the synthesis of N-Nitroso compounds, which are involved in epigenetic alterations and DNA-damage that can lead to the induction of cancer [241]. As another example, BCAA have been described to be altered in human diabetes, a risk factor for Alzheimer’s disease [242]. Preclinical studies have shown that the accumulation of these amino acids in brain promotes the phosphorylation of Tau proteins, which are involved in the development of Alzheimer [243]. Thus, the identification of alterations in these biomarkers and their precursors would be of high relevance.

**Figure 1:**
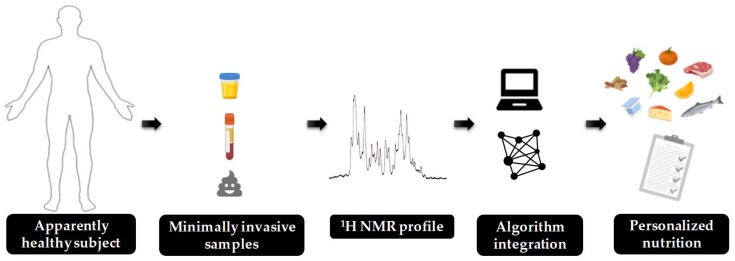
Pipeline explaining the steps that should be followed for an early detection of pre-diseases states and prevention of the development of cardiometabolic diseases through the ^1^H NMR (proton nuclear magnetic resonance) analysis of minimal invasive samples, thus getting metabolomics profile of the potential patients. Therefore, using this metabolomics information, we will be able to find a personalized interventional nutrition through the integration of studied algorithms to finally reduce or stop the development of the different cardiometabolic diseases.

Although the detection of molecular biomarkers by NMR techniques is very promising, there are several factors that must be taken into consideration. As a clear example, the selection of the analyzed biofluids is crucial. Blood and urine have been the preferred source of metabolites used by NMR analysis, but there are other useful and potential biofluids (Figure 1). One of them is feces, which might be a suitable biofluid for NMR analysis. In this case, the recollection is non-invasive and neither need a specialized person to acquire the biofluid via needle extraction. The challenge is to extract useful information from a complex sample that contains end products of human metabolism, different species of bacteria, end products from bacterial processes and epithelial cells from the colorectal mucosa via fecal NMR metabolomics [244]. Another fluid of interest, which has the same advantages as feces, is saliva, as it is also easy to obtain and it could inform about several metabolic processes. As an example, saliva biomarkers in AD early diagnostic were detected in a pilot study with NMR metabolomics. The development of accurate and sensitive salivary biomarkers would be ideal for screening those individuals at greatest risk of developing disease, translating the AD example to other diseases as MetS, obesity and T2D [245]. Thus, there is a need to promote the use of these promising biofluids to improve the detection of new biomarkers. 

Another factor that has a sharp influence on the detection and interpretation of new biomarkers is sample processing, which requires specific conditions depending on the analyzed biomarkers. As an example, several oxidative stress biomarkers, such as glutathione, are unstable and unreliable to detect by NMR due to its oxidation during sample processing. Moreover, other biomarkers such as acetate, can be easily overestimated in different biofluids because of the contamination of samples during its manipulation. Therefore, specific extraction and quantification procedures must be taken into consideration depending on the analyzed biomarkers and their chemical properties. 

Several molecular biomarkers involved in metabolic disorders have been excluded from this review as there is not enough evidence to be considered as biomarkers of early stages of disease. Despite the fact that further research is needed in order to enlarge the list of robust biomarkers exposed in this review, identification and aggrupation of early biomarkers in different risk factor clusters can be of great help to (a) make it easier to identify altered metabolic pathways when more than one early biomarker placed in the same cluster is changed; and (b) design personalized diets with ingredients that are described to target the identified metabolic alterations.

## 9. Conclusions

To sum up, from the identification and quantification of early biomarkers, different metabolic diseases could be treated in early states of the development of the diseases, before they could not be reversed. NMR metabolomics assessment is a reproducible and economic analysis of these metabolites which could be useful to detect these early disease development stages. This review summarizes some potential biomarkers that have been described in the literature related to different clusters which have been associated with metabolic diseases (carbohydrates metabolism, dyslipidemia, oxidative stress, inflammation, and gut microbiota), and have been used to achieve health information about the patients who may have symptoms related to metabolic disorders. If these biomarkers are assessed together instead of individually, the information obtained would be more complete and it would be a good strategy to detect cardiometabolic diseases in their early stages. However, the lack of qualitative analysis through NMR assessments take us to improve the methods used to process the samples and the way to analyze the recently known metabolites. Besides, it is necessary to find more metabolites related to these early stages of development of diseases, being characterized and intensively studied. When these further studies advance, we will be able to establish a fast and accurate method to prevent cardiometabolic and metabolic syndrome diseases in pre-stages of their development. 

## Figures and Tables

**Table 1:** Metabolomic biomarkers risk factors of metabolic syndrome (MetS) and related diseases by NMR approaches.

Biomarker	Level	Biofluid	Risk factor	Metabolic pathway	Pre-clinical evidences	Clinical evidences
Glucose	Increased	Serum, urine	Carbohydrate disruption	Glycolysis, gluconeogenesis, pyruvate metabolism	[25,26]	[19,27,28]
Lactate	Increased	Serum, urine	Carbohydrate disruption	Gluconeogenesis, Pyruvate metabolism	[29,30]	[27,31,32]
	Increased	Urine	Gut microbiota metabolism			[33]
Uric acid	Increased	Serum, urine, and renal extracts	Carbohydrate disruption	Purine metabolism	[34,35]	[36]
Propionylcarnitine	Increased	Plasma	Carbohydrate disruption	Lipid metabolism	-	[37,38,39,40]
Leucine (BCAA)	Increased	Serum/plasma, urine	Carbohydrate disruption	Amino acid metabolism	[25,26]	[32,41,42,43]
Isoleucine (BCAA)	Increased	Serum/plasma, urine	Carbohydrate disruption	Amino acid metabolism	[25,26]	[32,41,42,43,44]
Valine (BCAA)	Increased	Serum/plasma, urine	Carbohydrate disruption	Amino acid metabolism	[25,26]	[32,41,42,43,45,46]
Phenylalanine (AAA)	Increased	Serum/plasma, urine	Carbohydrate disruption	Amino acid metabolism	[25,26]	[32,41,42,43,47]
Tyrosine (AAA)	Increased	Serum/plasma, urine	Carbohydrate disruption	Amino acid metabolism	[25,26]	[32,41,42,43,44,48]
Glutamate	Increased	Serum	Carbohydrate disruption	Amino acid metabolism	[49]	[39,50,51]
Glutamine	Decreased	Serum, urine	Carbohydrate disruption	Amino acid metabolism	[30]	[39,50]
Citrate	Increased/decreased	Serum	Carbohydrate disruption	Tricarboxylic acid (TCA) cycle	[29,52]	[53]
TMAO	Increased	Plasma/Urine	Gut microbiota metabolism	Choline metabolism	[54]	[55,56]
Acetate	Increased	Plasma	Gut microbiota metabolism	Pyruvate metabolism	[57]	[58]
TMA	Increased/Decreased	Plasma/Urine	Gut microbiota metabolism	Choline metabolism	[59,60,61]	-
DMA	Increased/Decreased	Plasma/Urine	Gut microbiota metabolism	Choline metabolism	[59,62]	[27]
Succinate	Increased	Plasma	Gut microbiota metabolism	Succinate metabolism	[63]	[64]
NAG	Increased	Plasma/Serum	Inflammation pathway	Protein Glycosilation	-	[65,66,67]
LPCs	Increased	Plasma/Serum	Inflammation pathway	Phospholipid hydrolysis	-	[68]
SFA, MUFAs PUFAs: DHA, EPA/ ALA, AA	Decreased/Increased	Urine/Serum	Inflammation pathway	Lipid metabolism	[69]	-
		Serum	Dyslipidemia		[70]	[43]
3-hydroxybutirate	Increased	Urine/plasma	Dyslipidemia	Ketogenesis	[71]	[72]
Choline	Decreased	Serum	Dyslipidemia	Choline metabolism	[73,74]	[27]
Allantoin	Increased	Urine	Oxidative stress	Purine metabolism	[26,75,76,77]	-
Pseudouridine	Increased	Urine	Oxidative stress	Nucleic acid metabolism	-	[78,79,80]
Glycine	Decreased	Plasma/Serum	Oxidative stress	1C metabolism	-	[81,82]
Serine	Decreased	Plasma/Serum	Oxidative stress	1C metabolism	-	[81,82]

Branched chain aminoacids (BCAAs); aromatic aminoacids (AAAs); trimethylamine N-oxide (TMAO); trimethylamine (TMA); dimethylamine (DMA); lysophospholipids (LPCs); N-acetylglycoproteins (NAG); saturated fatty acids (SFA); monounsaturated fatty acids (MUFAs); polyunsaturated fatty acids (PUFAs); eicosapentaenoic acid (EPA); docosahexaenoic acid (DHA); arachidonic acid (ARA); arachidonic acid (AA).

**Table 2:** Standard levels of BCAAs and AAAs as essential amino acids in serum.

BCAAs	Valine	mmol/L	<0.2492	[140]
	Leucine	mmol/L	<0.1236	[141]
	Isoleucine	mmol/L	<0.0602	[141]
AAAs	Tyrosine	mmol/L	<0.0545	[142]
	Phenylalanine	mmol/L	<0.0781	[142]

**Table 3:** Metabolic pathways significantly affected by the proposed metabolites.

Pathway Name	Match Status	Metabolites Involved	FDR	Impact
Aminoacyl-tRNA biosynthesis	9/48	Phenylalanine; Glutamine; Glycine; Serine; Valine; Isoleucine; Leucine; Tyrosine; Glutamate;	1.4304 × 10^−6^	0.16667
Glyoxylate and dicarboxylate metabolism	6/32	Citrate; Serine; Glycine, Glutamate; Acetate; Glutamine	2.6791 × 10^−4^	0.1799
Valine, leucine and isoleucine biosynthesis	3/8	Leucine; Isoleucine; Valine	0.0055	0.0
Alanine, aspartate and glutamate metabolism	4/28	Glutamate; Glutamine; Citrate; Succinate	0.0175	0.3109
Phenylalanine, tyrosine and tryptophan biosynthesis	2/4	Phenylalanine; Tyrosine;	0.0208	1.0
Butanoate metabolism	3/15	3-Hydroxybutirate; Glutamate; Succinate	0.0208	0.0
Glutamine and glutamate metabolism	2/6	Glutamate; Glutamine	0.0378	0.5
Glutathione metabolism	3/28	Glutathione disulfide; Glycine; Glutamate;	0.0869	0.13537
Phenylalanine metabolism	2/10	Phenylalanine, Tyrosine	0.0873	0.35714

Adapted from the MetaboAnalyst results. Pathway name, match status (number of metabolites implicated in each pathway vs. the total implicated), metabolites involved, the False Discovery Rate (FDR) and Impact are shown in the table.
